# Physicians’ knowledge, attitude, and practice regarding the use of glucocorticoids in chronic obstructive pulmonary disease

**DOI:** 10.3389/fmed.2025.1583829

**Published:** 2025-09-30

**Authors:** Jingying Zhang, Xiaojing Lv, Xiaomin Lu, Yu Wei, Shi Chen

**Affiliations:** Department of Respiratory and Critical Care Medicine, Jiangsu Province Hospital of Chinese Medicine, Affiliated Hospital of Nanjing University of Chinese Medicine, Nanjing, China

**Keywords:** chronic obstructive pulmonary disease, glucocorticoids, physician, knowledge, attitude, practice, cross-sectional study

## Abstract

**Objective:**

This study aims to investigate physicians’ knowledge, attitude, and practice (KAP) regarding the use of glucocorticoids in the management of chronic obstructive pulmonary disease (COPD).

**Methods:**

A multicenter cross-sectional study was conducted from January to March 2024 in China, spearheaded by the Affiliated Hospital of Nanjing University of Chinese Medicine. A self-designed structured questionnaire was used to collect demographic data and assess participants’ KAP scores.

**Results:**

A total of 359 valid responses were included in the analysis. Of the participants, 211 (58.77%) were female, and 260 (72.42%) had attended relevant lectures or training. The mean scores for knowledge, attitude, and practice were 45.96 ± 7.80 (range: 11–55), 51.83 ± 5.14 (range: 12–60), and 32.39 ± 4.69 (range: 8–40), respectively. The Structural equation modeling showed that department (*β* = −3.25, *p* < 0.001), involvement in teaching (*β* = −2.93, *p* < 0.001), average number of COPD patients consulted (*β* = 1.28, *p* < 0.001), and training attendance (*β* = −3.42, *p* < 0.001) had a direct impact on knowledge. Knowledge (*β* = 0.29, *p* < 0.001) directly influenced attitude, while both knowledge (*β* = 0.29, *p* < 0.001) and attitude (*β* = 0.30, *p* = 0.014), along with training attendance (*β* = −1.52, *p* < 0.001), directly affected practice.

**Conclusion:**

Physicians demonstrated adequate knowledge, positive attitude, and proactive practice regarding glucocorticoid use in COPD management. Targeted educational programs, particularly for specific departments and teaching staff, are recommended to enhance knowledge and optimize glucocorticoid use in COPD treatment.

## Introduction

Chronic obstructive pulmonary disease (COPD) is a common lung disease characterized by airflow limitation and breathing difficulties. Symptoms include coughing, sometimes with sputum, shortness of breath, wheezing, and fatigue (1, 2). Globally, COPD is the third leading cause of death, responsible for 3.23 million deaths in 2019 (3). In China, COPD is also a significant public health issue, ranking as the third leading cause of death (4). By 2039, the prevalence of COPD in China is projected to reach 103.3 million, leading to a cumulative loss of 253.6 million quality-adjusted life years and 3.9 million excess deaths (5).

Management of COPD primarily involves the use of inhaled medications that promote bronchodilation and reduce inflammation, with regular, daily use recommended. These inhaled therapies can be combined with corticosteroids to further suppress airway inflammation. Glucocorticoids play a vital role in both the long-term management of COPD and the treatment of acute exacerbations, due to their potent anti-inflammatory properties (6, 7). However, inhaled corticosteroids (ICS) are associated with increased risks of oral candidiasis, hoarseness, skin bruising, and pneumonia (8), as well as a higher incidence of hip and upper extremity fractures when used in high doses (9). Additionally, glucocorticoid resistance is commonly observed in COPD, significantly reducing the anti-inflammatory efficacy of these medications (10).

The Knowledge, Attitude, and Practice (KAP) model suggests that behavior change is initiated through the acquisition of knowledge, which forms the foundation for developing attitudes and beliefs. These attitudes, in turn, drive the formation of practices and behaviors. However, knowledge alone is insufficient to induce behavioral change—it must first lead to a shift in attitudes and perceptions, which then prompts changes in behavior (11, 12). Physicians, as the primary prescribers and decision-makers in the management of COPD, play a critical role in the appropriate use of glucocorticoids. Their knowledge and attitude directly impact clinical decision-making and patient outcomes. Assessing their KAP can help identify areas for improvement, ensuring evidence-based and individualized care for COPD patients. Given their central role in COPD management, improving physicians’ practice in glucocorticoid use is crucial to enhancing patient outcomes.

Previous studies have shown that although glucocorticoids play an important role in COPD management, problems such as inappropriate use, increased risk of adverse effects, and glucocorticoid resistance remain (8–10). Guidelines provide detailed recommendations, but gaps between guidelines and real-world practice have been reported (13). KAP-related studies in other diseases also suggested that insufficient knowledge and inconsistent attitudes could affect practice (11, 12). However, few studies have focused on physicians’ KAP regarding glucocorticoid use in COPD.

Currently, the knowledge, attitude, and practice of physicians regarding the use of glucocorticoids in COPD treatment remain unclear. This study aims to investigate physicians’ knowledge, attitude, and practice regarding glucocorticoid use for COPD patients.

## Methods

### Study design and participants

This multicenter cross-sectional study was conducted from January to March 2024, led by the Affiliated Hospital of Nanjing University of Chinese Medicine, with participation from various centers across East China, involving physicians as study participants. Physicians were eligible if they: (1) held a valid medical license; (2) had experience diagnosing and treating COPD within the past year; and (3) understood the study’s purpose and voluntarily signed informed consent. Exclusion criteria were: (1) physicians in training, rotating, or interning. In addition, hospitals were eligible if they had the capability to independently diagnose and treat COPD and included departments such as respiratory medicine, general practice, internal medicine, geriatrics, or emergency medicine. Hospitals that lacked COPD diagnostic and treatment capacity, such as obstetrics and gynecology, dentistry, urology, and pediatrics, were excluded. This study was approved by the Ethics Committee of the Affiliated Hospital of Nanjing University of Chinese Medicine (Approval Number: 2023NL-067-02), and informed consent was obtained from all participants.

### Questionnaire

The questionnaire was developed based on established guidelines, including the European Respiratory Society guideline on ICS withdrawal in COPD (2020), the 2023 Canadian Thoracic Society Guideline on Pharmacotherapy in Patients with Stable COPD, and the 2021 Chinese expert consensus on the optimal use of glucocorticoids in COPD (14–16). The initial draft was reviewed by two respiratory specialists, each with over 20 years of professional experience. Revisions were made according to the experts’ feedback, which included the removal of redundant questions and ambiguous wording to ensure the content validity of the questionnaire. A preliminary survey involving 42 participants was conducted, followed by reliability testing. The overall Cronbach’s *α* coefficient was 0.903, indicating strong internal consistency, with values of 0.9509 for the knowledge section, 0.7660 for the attitude section, and 0.7814 for the practice section.

The final version of the questionnaire, administered in Chinese, consisted of four sections: demographic information (including age, gender, education level, department, years of work experience, professional title, hospital type and level, involvement in teaching and research, weekly COPD patient consultations, and experience with relevant training or lectures), and three KAP dimensions—knowledge, attitude, and practice. All sections were measured using a five-point Likert scale. The knowledge section comprised 11 items, with scores ranging from 5 (“very knowledgeable”) to 1 (“completely unclear”), for a total score range of 11–55. The attitude section included 12 items, scored from 5 (“strongly agree”) to 1 (“strongly disagree”), with a total score range of 12–60. The practice section had 8 items, scored from 5 (“always”) to 1 (“never”), resulting in a total score range of 8–40. A score threshold of ≥70.0% was used to define adequate knowledge, positive attitude, and proactive practice (17, 18). Additionally, the questionnaire included a common knowledge trap question to filter out invalid responses from participants who answered without thoroughly reviewing the items. The English version of the questionnaire is provided in the Appendix. Questionnaire.

### Questionnaire distribution and quality control

Prior to the study, 86 eligible hospitals in East China were contacted, and 75 institutions agreed to participate. The study utilized electronic questionnaires for data collection. Data collection was conducted online using an electronic questionnaire. The online questionnaire was created via the Wenjuanxing platform and a QR code for the survey was generated. The QR code was distributed to participants at the hospitals through WeChat for data collection. During the questionnaire completion process, research assistants provided clarifications to ensure that participants fully understood the questionnaire’s content and the objectives of the study. After data collection, members of the research team conducted quality checks. Questionnaires were considered invalid and excluded if the completion time was less than 90 s, if trap questions were answered incorrectly, or if logical inconsistencies or repeated patterns in responses were detected. A threshold of 90 s was applied because a shorter duration was insufficient to carefully read and complete all items, suggesting a high likelihood of careless or invalid responses.

### Model and hypotheses

**Figure 1:**
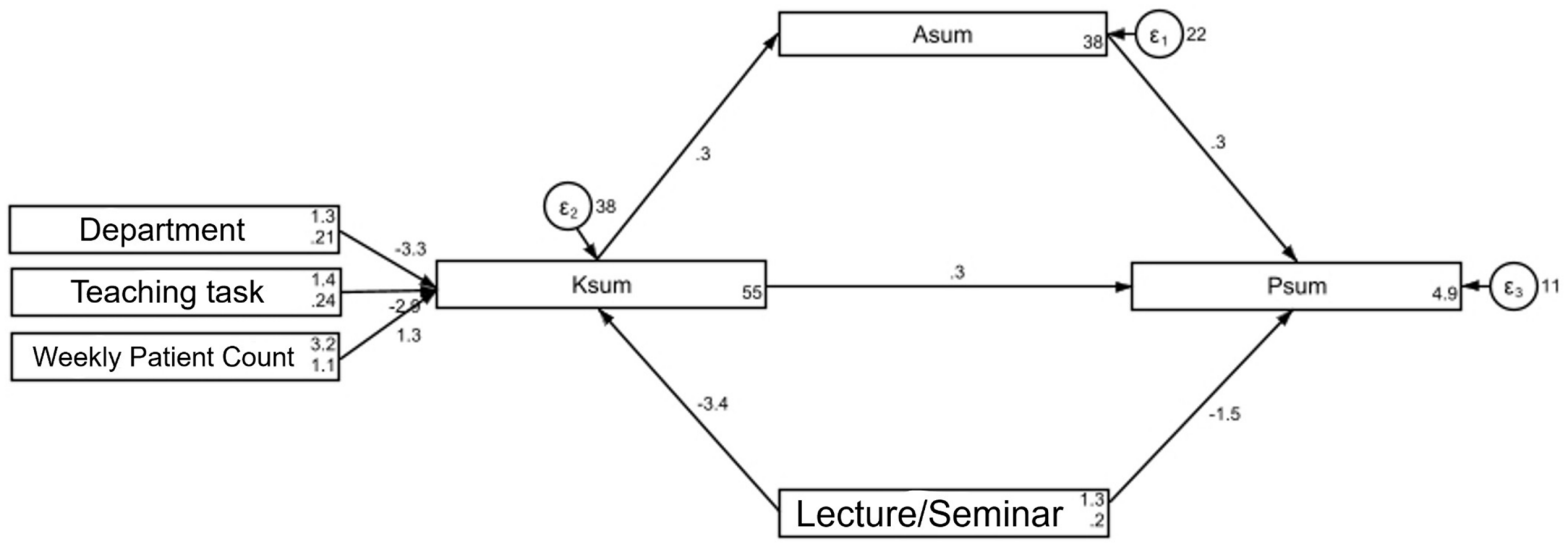
SEM analysis.

Based on the knowledge-attitude-practice (KAP) theoretical framework, a structural equation model (SEM) was constructed to explore the interactions among knowledge, attitude, and practice regarding glucocorticoid use in COPD. Three hypotheses were proposed: (H1) knowledge directly affects attitude, (H2) knowledge directly affects practice, and (H3) knowledge indirectly affects practice through attitude. Figure 1 presents the conceptual model and hypothesized paths.

### Statistical analysis

Statistical analyses were performed using SPSS 26.0 (IBM, Armonk, NY, United States) and AMOS 24.0 (IBM, Armonk, NY, United States). Continuous variables were reported as mean ± SD for normally distributed variables, and median with interquartile range for non-normally distributed variables. Normality tests were conducted to determine appropriate statistical methods. For two-group comparisons, the t-test was used for normally distributed data, while the Wilcoxon Mann–Whitney test was applied to non-normally distributed data. ANOVA was used for three or more groups with normal distribution and homogeneity of variance, and the Kruskal-Wallis test for non-normal data. Spearman correlation analysis was used to evaluate the relationships between knowledge, attitude, and practice scores. Model fit was assessed using the following criteria: root mean square error of approximation (RMSEA) < 0.08, standardized root mean square residual (SRMR) < 0.08, Tucker-Lewis index (TLI) > 0.80, and comparative fit index (CFI) > 0.80. A two-sided *p*-value < 0.05 was considered statistically significant.

## Results

### Demographic information and KAP scores

A total of 410 questionnaires were initially collected. After excluding 25 responses with completion times less than 90 s, 4 with incorrect age entries, 15 reporting ‘0’ COPD patient consultations per week, and 11 with incorrect answers to trap questions, 359 valid responses were analyzed. Among the participants, 211 (58.77%) were female, 178 (49.58%) were aged 35–44 years, 164 (45.68%) held a master’s degree, 143 (39.83%) had senior professional titles (including associate senior titles), 268 (74.65%) worked in tertiary hospitals, 212 (59.05%) were involved in teaching, 180 (50.14%) were engaged in research, 120 (33.43%) consulted 6–15 COPD patients per week, and 260 (72.42%) had attended relevant lectures or training. The mean ± SD scores for knowledge, attitude, and practice were 45.96 ± 7.80 (range: 11–55), 51.83 ± 5.14 (range: 12–60), and 32.39 ± 4.69 (range: 8–40), respectively. Demographic analysis revealed that knowledge and practice scores were significantly associated with age (*p* < 0.001 for both), department (*p* < 0.001 and *p* = 0.001), years of work experience (*p* < 0.001 for both), professional title (*p* < 0.001 for both), hospital level (*p* = 0.015 and *p* = 0.021), involvement in teaching (*p* < 0.001 and *p* = 0.003), and the average number of COPD patients consulted per week (*p* < 0.001 and *p* = 0.001). Furthermore, knowledge scores were significantly associated with involvement in research (*p* = 0.010). Attendance at relevant lectures or training was significantly associated with higher knowledge, attitude, and practice scores (*p* < 0.001, *p* = 0.036, *p* < 0.001; Table 1).

**Table 1:** Baseline information and KAP scores.

*N* = 359	*N* (%)	Knowledge score		Attitude score		Practice score	
		Mean ± SD	*p*	Mean ± SD	*p*	Mean ± SD	*p*
Total score		45.96 ± 7.80		51.83 ± 5.14		32.39 ± 4.69	
Gender			0.400		0.193		0.698
Male	148 (41.23)	46.5 ± 6.51		52.34 ± 5.10		32.57 ± 4.71	
Female	211 (58.77)	45.5 ± 7.47		51.47 ± 5.15		32.26 ± 4.66	
Age (years)			**<0.001**		0.085		**<0.001**
<35	115 (32.03)	43.4 ± 6.83		51.08 ± 5.06		30.85 ± 4.58	
35 ~ 44	178 (49.58)	46.7 ± 6.97		52.32 ± 5.24		32.98 ± 4.70	
≥45	66 (18.38)	48.2 ± 6.73		51.80 ± 4.92		33.45 ± 4.16	
Education level			0.3677		0.547		0.403
Associate’s/bachelor’s degree	142 (39.55)	45.3 ± 7.87		52.15 ± 5.61		32.66 ± 4.70	
Master’s degree	164 (45.68)	45.9 ± 6.70		51.79 ± 4.81		32.08 ± 4.55	
Doctorate	53 (14.76)	47.4 ± 5.90		51.09 ± 4.81		32.60 ± 5.07	
Department			**<0.001**		0.880		**0.001**
Respiratory medicine	255 (71.03)	47.4 ± 6.32		51.81 ± 4.93		32.97 ± 4.30	
Other	104 (28.97)	42.2 ± 7.54		51.86 ± 5.64		30.96 ± 5.27	
Years of work experience			**<0.001**		0.536		**<0.001**
≤5 years	64 (17.83)	43.9 ± 6.80		51 ± 4.99		31.17 ± 4.71	
5 ~ 10 years	100 (27.86)	43.8 ± 7.21		52.23 ± 5.31		31.15 ± 4.84	
11–20 years	130 (36.21)	47.2 ± 6.93		51.86 ± 5.23		33.34 ± 4.58	
≥21 years	65 (18.11)	48.6 ± 6.04		51.96 ± 4.83		33.58 ± 3.88	
Professional title			**<0.001**		0.172		**<0.001**
Junior Title or Below	79 (22.01)	42.4 ± 6.85		50.93 ± 5.09		30.65 ± 4.61	
Intermediate Title	137 (38.16)	45.5 ± 7.34		52.14 ± 5.45		32.19 ± 4.79	
Senior Title (including Associate Senior)	143 (39.83)	48.3 ± 6.05		52.02 ± 4.83		33.53 ± 4.31	
Hospital type			0.621		0.884		0.450
Public Hospital	312 (86.91)	46.0 ± 7.03		51.84 ± 5.09		32.32 ± 4.63	
Private Hospital	47 (13.09)	45.2 ± 7.55		51.76 ± 5.49		32.85 ± 5.03	
Hospital level			**0.015**		0.343		**0.021**
Primary Hospital	27 (7.52)	41.6 ± 9.26		50.33 ± 5.71		29.51 ± 6.31	
Secondary hospital	64 (17.83)	45.4 ± 7.32		52.48 ± 5.60		33.07 ± 4.70	
Tertiary hospital	268 (74.65)	46.5 ± 6.65		51.82 ± 4.95		32.51 ± 4.40	
Involvement in teaching			**<0.001**		0.697		**0.003**
Yes	212 (59.05)	47.5 ± 6.18		51.92 ± 4.84		33.09 ± 4.20	
No	147 (40.95)	43.6 ± 7.67		51.70 ± 5.55		31.37 ± 5.15	
Involvement in research			**0.010**		0.662		0.095
Yes	180 (50.14)	47.0 ± 6.31		51.91 ± 4.81		32.87 ± 4.20	
No	179 (49.86)	44.8 ± 7.67		51.74 ± 5.46		31.89 ± 5.08	
Average number of COPD patients consulted per week in the past year			**<0.001**		0.631		**0.001**
1–5	115 (32.03)	42.6 ± 8.02		51.36 ± 5.18		30.98 ± 5.15	
6–15	120 (33.43)	46.6 ± 5.96		51.92 ± 5.13		32.61 ± 4.23	
16–30	65 (18.11)	48.1 ± 6.14		52.01 ± 5.24		33.4 ± 4.42	
31 or more	59 (16.43)	48.6 ± 5.87		52.35 ± 5.03		33.55 ± 4.27	
Participation in COPD management or glucocorticoid use related lectures or training (online or offline) in the past year			**<0.001**		**0.036**		**<0.001**
Yes	260 (72.42)	47.2 ± 6.36		52.20 ± 5.15		33.30 ± 3.91	
No	99 (27.58)	42.6 ± 7.83		50.85 ± 4.99		29.98 ± 5.63	

SD, standard deviation; COPD, chronic obstructive pulmonary disease; ICS, inhaled corticosteroids; SCS, systemic corticosteroids.The bold values indicate statistical significance.

### Distribution of responses to knowledge, attitude, and practice

The distribution of responses in the knowledge dimension revealed that the highest percentages of participants selecting “Heard of it” and “Slightly familiar” were for the items “Strategies for Reducing and Discontinuing ICS” (K10) with 18.66%, “The Impact of Eosinophil Count on Glucocorticoid Treatment in COPD Patients” (K8) with 18.11%, and “Strategies for Reducing and Discontinuing SCS” (K11) with 13.93% (Table 2). In addition, very few participants selected the “completely unclear” option across the knowledge items (≤ 0.3%). However, notable knowledge gaps were identified. The highest percentage of less-than-ideal responses (indicating a lack of familiarity) was found for Item K8 (Impact of eosinophil count), with 18.67% of respondents, followed by Item K10 (Strategies for discontinuing ICS) (18.66%), and Item K6 (Potential side effects of long-term ICS use) (13.09%).

**Table 2:** Knowledge dimension distribution.

*N* (%)	Very familiar	Fairly familiar	Heard of it	Slight familiar	Completely unclear
1. The Mechanism of Action of Glucocorticoids in the Treatment of COPD.	111 (30.92)	220 (61.28)	18 (5.01)	9 (2.51)	1 (0.28)
2. Differences in the Role of Inhaled Glucocorticoids (ICS) and Systemic Glucocorticoids (SCS) in COPD Treatment.	112 (31.2)	212 (59.05)	26 (7.24)	9 (2.51)	/
3. Timing and Indications for the Use of ICS.	133 (37.05)	191 (53.2)	22 (6.13)	12 (3.34)	1 (0.28)
4. Timing and Indications for the Use of SCS.	120 (33.43)	202 (56.27)	22 (6.13)	15 (4.18)	/
5. Timing, Dosage, and Duration of Glucocorticoid Use During Acute Exacerbations of COPD.	125 (34.82)	191 (53.2)	26 (7.24)	17 (4.74)	/
6. Potential Systemic Side Effects of Long-Term Use of ICS.	122 (33.98)	190 (52.92)	28 (7.8)	18 (5.01)	1 (0.28)
7. Potential Systemic Side Effects of Long-Term Use of SCS.	140 (39)	197 (54.87)	16 (4.46)	6 (1.67)	/
8. The Impact of Eosinophil Count on Glucocorticoid Treatment in COPD Patients.	118 (32.87)	174 (48.47)	35 (9.75)	30 (8.36)	2 (0.56)
9. Correct Usage Methods and Precautions for ICS.	143 (39.83)	188 (52.37)	20 (5.57)	8 (2.23)	/
10. Strategies for Reducing and Discontinuing ICS.	107 (29.81)	185 (51.53)	38 (10.58)	29 (8.08)	/
11. Strategies for Reducing and Discontinuing SCS.	113 (31.48)	195 (54.32)	33 (9.19)	17 (4.74)	1 (0.28)

COPD, chronic obstructive pulmonary disease; ICS, inhaled corticosteroids; SCS, systemic corticosteroids.

In the attitude dimension, 11.42% disagreed with the necessity of ICS in treating stable COPD (A1), 6.41% disagreed with the use of SCS for acute exacerbations (A2), and 18.94% disagreed that the risks of long-term glucocorticoid use outweigh the benefits (A7) (Table 3). Moreover, “strongly disagree” responses were rare, generally ≤1%. The highest percentage of neutral, disagree, or strongly disagree responses was observed for Item A1 (Necessity of ICS in stable COPD) at 23.96%, and Item A2 (Necessity of SCS in acute exacerbations) at 18.95%. Furthermore, a significant portion of respondents expressed concern about the risks of long-term use, with 79.67% agreeing or feeling neutral that the risks may outweigh the benefits.

**Table 3:** Attitude dimension distribution.

*N* (%)	Strongly agree	Agree	Neutral	Disagree	Strongly disagree
1. I believe that ICS are necessary in the treatment of stable COPD.	131 (36.49)	142 (39.55)	43 (11.98)	41 (11.42)	2 (0.56)
2. I believe that SCS are necessary in the treatment of acute exacerbations of COPD.	123 (34.26)	168 (46.8)	42 (11.7)	23 (6.41)	3 (0.84)
3. I believe that the use of glucocorticoids should strictly follow guidelines and expert consensus.	172 (47.91)	169 (47.08)	17 (4.74)	1 (0.28)	/
4. I believe that it is necessary to regularly assess the ICS treatment regimen for COPD patients.	199 (55.43)	144 (40.11)	12 (3.34)	4 (1.11)	/
5. I support adjusting the use of ICS based on eosinophil count.	133 (37.05)	182 (50.7)	39 (10.86)	5 (1.39)	/
6. I believe that ICS use should be gradually reduced or stopped in patients without clear indications.	135 (37.6)	200 (55.71)	17 (4.74)	7 (1.95)	/
7. I believe that the risks of long-term glucocorticoid use outweigh the benefits for COPD patients.	79 (22.01)	140 (39)	67 (18.66)	68 (18.94)	5 (1.39)
8. I believe that individualized treatment for COPD patients is very important.	226 (62.95)	127 (35.38)	6 (1.67)	/	/
9. I believe that there are misconceptions about the effects of glucocorticoids, especially regarding short-term and long-term adverse reactions.	165 (45.96)	176 (49.03)	16 (4.46)	2 (0.56)	
10. I believe that patient education is a crucial component in the use of both inhaled and systemic glucocorticoids.	214 (59.61)	141 (39.28)	3 (0.84)	1 (0.28)	/
11. I believe that collaboration among healthcare teams is very important in managing glucocorticoid use in COPD patients.	205 (57.1)	151 (42.06)	3 (0.84)	/	/
12. I believe that hospitals or departments should offer more educational seminars on the use of glucocorticoids in COPD.	198 (55.15)	157 (43.73)	3 (0.84)	1 (0.28)	/

COPD, chronic obstructive pulmonary disease; ICS, inhaled corticosteroids; SCS, systemic corticosteroids.

In the practice dimension, 27.29% sometimes or rarely reassessed the glucocorticoid treatment regimen for COPD patients (P1), 33.42% sometimes or rarely adjusted ICS use based on the patient’s eosinophil count (P3), and 27.02% sometimes or rarely used SCS routinely during acute exacerbations (P4) (Table 4). Similarly, “never” responses in the practice items were uncommon, generally below 1%. The least frequent practice was Item P3 (Adjusting ICS use based on eosinophil count), where 34.53% of respondents indicated doing so only “sometimes,” “rarely,” or “never.” This was followed by Item P1 (Regularly reassessing the glucocorticoid regimen) at 28.13%, and Item P4 (Routinely using SCS during acute exacerbations) at 27.3%, highlighting specific areas where clinical practice may not consistently align with guideline recommendations.

**Table 4:** Practice dimension distribution.

*N* (%)	Always	Often	Sometimes	Rarely	Never
1. I regularly reassess the glucocorticoid treatment regimen for COPD patients.	76 (21.17)	182 (50.7)	80 (22.28)	18 (5.01)	3 (0.84)
2. I individualize glucocorticoid treatment for COPD patients according to the latest guidelines and expert consensus.	99 (27.58)	186 (51.81)	55 (15.32)	18 (5.01)	1 (0.28)
3. I adjust the use of ICS based on the patient’s eosinophil count.	80 (22.28)	155 (43.18)	81 (22.56)	39 (10.86)	4 (1.11)
4. I routinely use SCS during acute exacerbations of COPD.	83 (23.12)	178 (49.58)	77 (21.45)	20 (5.57)	1 (0.28)
5. I provide detailed instructions and precautions to COPD patients regarding the use of ICS.	163 (45.4)	162 (45.13)	28 (7.8)	4 (1.11)	2 (0.56)
6. I proactively educate patients and their families about the potential risks of glucocorticoid use in COPD treatment.	124 (34.54)	169 (47.08)	50 (13.93)	15 (4.18)	1 (0.28)
7. I closely monitor for any unusual conditions or adverse reactions during the use of medications by patients.	135 (37.6)	190 (52.92)	27 (7.52)	7 (1.95)	/
8. I regularly update my knowledge by studying the latest guidelines and expert consensus.	119 (33.15)	186 (51.81)	45 (12.53)	9 (2.51)	/

COPD, chronic obstructive pulmonary disease; ICS, inhaled corticosteroids; SCS, systemic corticosteroids.

### Correlations among KAP

Correlation analysis revealed positive associations between knowledge and attitude scores (*r* = 0.4207, *p* < 0.001), and between knowledge and practice scores (*r* = 0.6248, *p* < 0.001). Attitude scores were also positively correlated with practice scores (*r* = 0.5504, *p* < 0.001; Table 5).

**Table 5:** Correlation analysis.

Dimensions	Knowledge dimension	Attitude	Practice
Knowledgedimension	1		
Attitude	0.4207 (P < 0.001)	1	
Practice	0.6248 (P < 0.001)	0.5504 (P < 0.001)	1

KAP, knowledge, attitude, and practice.

### Interactions between KAP

The structural equation model showed good fit (RMSEA = 0.059, SRMR = 0.034; TLI = 0.957; CFI = 0.980), indicating the model adequately captured the relationships between variables (Supplementary Table S1). And the specific effects of the factors on KAP were detailed in Supplementary Table S2. Specifically, department (*β* = −3.25, *p* < 0.001), involvement in teaching (*β* = −2.93, *p* < 0.001), average number of COPD patients consulted (*β* = 1.28, *p* < 0.001), and attendance at lectures or training (*β* = −3.42, *p* < 0.001) directly influenced knowledge. Knowledge (*β* = 0.29, *p* < 0.001) had a direct effect on attitude. Both knowledge (*β* = 0.29, *p* < 0.001) and attitude (*β* = 0.30, *p* = 0.014), as well as lectures or training (*β* = −1.52, *p* < 0.001), directly influenced practice. Additionally, department (*β* = −0.97, *p* < 0.001), involvement in teaching (*β* = −0.87, *p* < 0.001), average number of COPD patients consulted (*β* = 0.38, *p* < 0.001), and lectures or training (*β* = −1.02, *p* < 0.001) indirectly influenced attitude. Knowledge (*β* = 0.09, *p* < 0.001), department (*β* = −1.26, *p* < 0.001), involvement in teaching (*β* = −1.14, *p* < 0.001), the average number of COPD patients consulted (*β* = 0.50, *p* < 0.001), and lectures or training (*β* = −1.33, *p* < 0.001) indirectly influenced practice (Supplementary Table S3; Figure 1).

## Discussion

Physicians demonstrated adequate knowledge, positive attitude, and proactive practice regarding the use of glucocorticoids in the management of COPD, with correlations between KAP scores and the results of SEM indicating that improvements in knowledge can lead to better attitude and practice.

These findings are consistent with previous research, which showed high awareness of COPD management guidelines among primary care and respiratory specialists across 12 countries; however, gaps were observed in the application of these guidelines to treatment recommendations (13).

In terms of the relationship between KAP dimensions, both correlation analyses and SEM results confirmed significant associations between knowledge, attitude, and practice scores. The positive correlation between knowledge and practice, as well as between attitude and practice, indicates that improved knowledge is likely to lead to better clinical practice, as supported by previous literature (19, 20). Furthermore, SEM analysis showed that knowledge directly influenced both attitude and practice, emphasizing the critical role that enhancing knowledge plays in shaping positive attitude and behaviors toward COPD management. This finding reinforces the need for continued education and training programs to ensure sustained improvement in clinical practice related to glucocorticoid use.

Significant differences in knowledge and practice scores were observed across several demographic variables. For example, physicians in respiratory medicine departments had significantly higher knowledge and practice scores compared to those in other departments, a finding corroborated by SEM results. This suggests that specialized experience contributes to greater competence in managing chronic disease, a conclusion also reflected in previous studies (21, 22). Similarly, those involved in teaching and with more years of work experience exhibited higher knowledge and practice scores, further supported by SEM results. These findings highlight the importance of continuous professional development and mentorship in improving COPD care.

However, it is notable that no significant differences were found in attitude scores across most demographic variables. This may suggest that, while knowledge and practice vary by professional experience, attitude toward glucocorticoid use remain consistently positive across different subgroups. One possible explanation is that attitude are more influenced by broader institutional guidelines and training programs, which ensure uniformity in treatment perspectives (23, 24).

The knowledge results show that physicians generally possess a solid understanding of glucocorticoid use in COPD, with the majority being familiar with key aspects such as the mechanism of action, differences between ICS and SCS, and the potential systemic side effects of long-term glucocorticoid use. However, some areas showed less familiarity, such as the impact of eosinophil count on treatment decisions and strategies for reducing or discontinuing ICS, where over 10% of participants reported only having heard of or being slightly familiar with these topics. This indicates that misconceptions still exist among a subgroup of physicians, which should be addressed through targeted education. Similar studies have also highlighted gaps in specific areas of glucocorticoid knowledge, suggesting that these deficiencies may contribute to suboptimal treatment outcomes (25, 26). To address these gaps, more focused educational programs could be developed, targeting the lesser-known aspects of glucocorticoid use, such as individualized treatment adjustments based on eosinophil count and long-term management strategies. These programs could be delivered through practical workshops and case studies, ensuring that physicians can apply this knowledge in clinical settings (27, 28).

Physicians generally demonstrated positive attitude toward the use of glucocorticoids in COPD management, particularly in supporting individualized treatment and regular reassessment of ICS regimens. However, there was some hesitancy regarding the necessity of ICS in stable COPD and the risks associated with long-term glucocorticoid use, where a notable proportion of participants expressed disagreement or uncertainty. This mirrors findings in other studies, where attitude toward long-term glucocorticoid use often reflect concerns over side effects, potentially leading to cautious or inconsistent prescribing practice (29–32).

While most physicians reported regularly reassessing glucocorticoid regimens and individualizing treatment, certain practice—such as adjusting ICS use based on eosinophil count and educating patients about glucocorticoid risks—were less consistently performed. A significant proportion of physicians only “sometimes” or “rarely” engaged in these practice, indicate challenges in implementing evidence-based adjustments in routine practice. To improve these areas, more structured protocols could be introduced to ensure routine consideration of eosinophil count in treatment decisions. Additionally, departments could introduce checklists or standardized patient education materials to ensure that key information about the risks and proper use of glucocorticoids is consistently communicated. Furthermore, encouraging more frequent monitoring and feedback on practice habits, possibly through peer review systems or patient outcome tracking, could further reinforce positive practice (33, 34).

Limitations of this study include the reliance on self-reported questionnaires, which may introduce response bias and affect the accuracy of the data. Additionally, the cross-sectional design limits the ability to establish causal relationships between demographic factors and KAP scores. Besides, the study was conducted in a specific region of China, which may limit the generalizability of the findings to other geographic areas or healthcare systems. Finally, this study did not assign weights to individual questionnaire items, and potential interactions between questions were not considered. While this is consistent with most KAP surveys, it may limit the ability to reflect the relative importance of different factors.

In conclusion, this study found that physicians generally exhibited adequate knowledge, positive attitude, and proactive practice regarding the use of glucocorticoids in treating COPD, with key demographic factors such as department and teaching involvement influencing their KAP scores. To enhance clinical outcomes, targeted educational programs and training sessions should be developed to address specific gaps in knowledge and practice, particularly for physicians in departments with lower KAP scores.

## Data Availability

The original contributions presented in the study are included in the article/Supplementary material, further inquiries can be directed to the corresponding author/s.
