# Supplementary material for: Physicians’ knowledge, attitude, and practice regarding the use of glucocorticoids in chronic obstructive pulmonary disease
Source: Front Med (Lausanne). 2025 Sep 30;12:1583829. doi: 10.3389/fmed.2025.1583829 (PMC12518364; doi:10.3389/fmed.2025.1583829)
Supplement: Supplementary file 1 [file Table_1.DOCX]

**Table S1. SEM model fit**

| Indicators | Reference | Results |
| --- | --- | --- |
| RMSEA | <0.08 Good | 0.059 |
| SRMR | <0.08 Good | 0.034 |
| TLI | >0.8 Good | 0.957 |
| CFI | >0.8 Good | 0.980 |

**Table S2. SEM Results**

|  |  | Estimate | P>\|z\| |
| --- | --- | --- | --- |
|  |  |  |  |
| Asum |  |  |  |
|  | Ksum | 0.298 | <0.001 |
| Psum |  |  |  |
|  | Asum | 0.302 | <0.001 |
|  | Ksum | 0.299 | <0.001 |
|  | Lectures or training | -1.527 | <0.001 |
| Ksum |  |  |  |
|  | Department | -3.252 | <0.001 |
|  | Involvement in Teaching | -2.937 | <0.001 |
|  | Average number of COPD patients consulted | 1.289 | <0.001 |
|  | Lectures or training | -3.428 | <0.001 |

**Table S3. Analysis of direct and indirect effects**

| Model paths |  | Total effects | | Direct Effect | | Indirect effect | |
| --- | --- | --- | --- | --- | --- | --- | --- |
|  |  | β (95% CI) | P | β (95% CI) | P | β (95% CI) | P |
| Asum |  |  |  |  |  |  |  |
|  | Ksum | 0.29 (0.23, 0.36) | <0.001 | 0.29 (0.23, 0.36) | <0.001 | —— | —— |
|  | Department | -0.97 (-1.46, -0.47) | <0.001 | —— | —— | -0.97 (-1.46, -0.47) | <0.001 |
|  | Involvement in Teaching | -0.87 (-1.31, -0.43) | <0.001 | —— | —— | -0.87 (-1.31, -0.43) | <0.001 |
|  | Average number of COPD patients consulted | 0.38 (0.17, 0.59) | <0.001 | —— | —— | 0.38 (0.17, 0.59) | <0.001 |
|  | Lectures or training | -1.02 (-1.51, -0.52) | <0.001 | —— | —— | -1.02 (-1.51, -0.52) | <0.001 |
| Psum |  |  |  |  |  |  |  |
|  | Asum | 0.30 (0.22, 0.37) | <0.001 | 0.30 (0.22, 0.37) | <0.001 | —— | —— |
|  | Ksum | 0.38 (0.33, 0.44) | <0.001 | 0.29 (0.24, 0.35) | <0.001 | 0.09 (0.060, 0.120) | <0.001 |
|  | Department | -1.26 (-1.87, -0.66) | <0.001 | —— | —— | -1.26 (-1.87, -0.66) | <0.001 |
|  | Involvement in Teaching | -1.14 (-1.68, -0.60) | <0.001 | —— | —— | -1.14 (-1.68, -0.60) | <0.001 |
|  | Average number of COPD patients consulted | 0.50 (0.24, 0.75) | <0.001 | —— | —— | 0.50 (0.24, 0.75) | <0.001 |
|  | Lectures or training | -2.86 (-3.81, -1.91) | <0.001 | -1.52 (-2.31, -0.73) | <0.001 | -1.33 (-1.93, -0.73) | <0.001 |
| Ksum |  |  |  |  |  |  |  |
|  | Department | -3.25 (-4.73, -1.76) | <0.001 | -3.25 (-4.73, -1.76) | <0.001 | —— | —— |
|  | Involvement in Teaching | -2.93 (-4.25, -1.62) | <0.001 | -2.93 (-4.25, -1.62) | <0.001 | —— | —— |
|  | Average number of COPD patients consulted | 1.28 (0.65, 1.92) | <0.001 | 1.28 (0.65, 1.92) | <0.001 | —— | —— |
|  | Lectures or training | -3.42 (-4.88, -1.97) | <0.001 | -3.42 (-4.88, -1.97) | <0.001 | —— | —— |
