# Supplementary material for: Physicians’ knowledge, attitude, and practice regarding the use of glucocorticoids in chronic obstructive pulmonary disease
Source: Front Med (Lausanne). 2025 Sep 30;12:1583829. doi: 10.3389/fmed.2025.1583829 (PMC12518364; doi:10.3389/fmed.2025.1583829)
Supplement: Supplementary file 2 [file Table_2.DOCX]

| Questionnaire ID: |
| --- |
| Dear Participant,  We are researchers from the hospital and sincerely invite you to participate in our study. This research aims to understand physicians’ knowledge, attitudes, and practices regarding the use of glucocorticoids in chronic obstructive pulmonary disease (COPD). The findings will provide evidence for developing scientific intervention strategies, which may help improve the health of more people in the future. Your participation in this study is voluntary. The study has been reviewed and approved by the Ethics Committee. If you agree to participate, please read the following instructions carefully:  1. Please complete the questionnaire. There are no right or wrong answers; simply respond according to your actual situation. You may raise any questions during the process, and after completion, please submit the questionnaire promptly.  2. This study only involves a simple questionnaire survey. It will not cause harm to your physical or psychological health. However, it will include some personal information, such as your gender and age. We will strictly ensure confidentiality and will not disclose your information. Please feel assured when filling it out.  3. As a participant, you may request information about the study and its progress at any time. If you decide to withdraw, please inform us, and your data will not be included in the research results.  Finally, we sincerely thank you for taking the time to support our scientific research!  □I have been informed and agree that the data collected will be used for scientific research.  Signature of Informed Consent:  Date of Participation: ______ Year ______ Month ______ Day |

| **Part 1 Basic Information** | | | |
| --- | --- | --- | --- |
| 1. **Your gender:** | a. Male b. Female | | |
| 1. **Your age: ____ years (please enter an integer in the online questionnaire)** | | |  |
| 1. **Your educational level:** | a. Associate degree b. Bachelor’s degree c. Master’s degree d. Doctoral degree | | |
| 1. **Your department:** | a. Respiratory Medicine b. Internal Medicine c. General Practice d. Emergency Medicine e. Geriatrics f. Other | | |
| 1. **Your years of work experience:** | a. ≤ 5 years b. 5–10 years c. 11–20 years d. ≥ 21 years | | |
| 1. **Your professional title:** | a. Junior title or below b. Intermediate title c. Senior title (including associate senior) | | |
| 1. **Type of hospital you work in:** | a. Public hospital b. Private hospital | | |
| 1. **Level of hospital you work in:** | a. Primary hospital b. Secondary hospital c. Tertiary hospital | | |
| 1. **Do you undertake teaching responsibilities?** | | a. Yes b. No | |
| 1. **Do you undertake scientific research responsibilities?** | | a. Yes b. No | |
| 1. **On average, how many COPD patients do you see per week in the past year?** | | a. 0 b. 1–5 c. 6–15 d. 16–30 e. ≥ 31 | |
| 1. **In the past year, have you attended any lectures or training (online or offline) related to COPD management or glucocorticoid use?** | | a. Yes b. No | |

| **Part 2 Knowledge about the Use of Glucocorticoids in COPD** | | | | | |  |
| --- | --- | --- | --- | --- | --- | --- |
| **For the following statements, please indicate your level of understanding:** | | | | | | |
| 1. **Mechanism of action of glucocorticoids in the treatment of COPD.** | Very familiar | Fairly familiar | Heard of it | Slightly familiar | Not familiar at all | |
| 1. **Different roles of inhaled corticosteroids (ICS) and systemic corticosteroids (SCS) in the treatment of COPD.** | Very familiar | Fairly familiar | Heard of it | Slightly familiar | Not familiar at all | |
| 1. **Timing and indications for the use of inhaled corticosteroids (ICS).** | Very familiar | Fairly familiar | Heard of it | Slightly familiar | Not familiar at all | |
| 1. **Timing and indications for the use of systemic corticosteroids (SCS).** | Very familiar | Fairly familiar | Heard of it | Slightly familiar | Not familiar at all | |
| 1. **Timing, dosage, and duration of glucocorticoid use during acute exacerbations of COPD.** | Very familiar | Fairly familiar | Heard of it | Slightly familiar | Not familiar at all | |
| 1. **Potential systemic side effects of long-term use of inhaled corticosteroids (ICS).** | Very familiar | Fairly familiar | Heard of it | Slightly familiar | Not familiar at all | |
| 1. **Potential systemic side effects of long-term use of systemic corticosteroids (SCS).** | Very familiar | Fairly familiar | Heard of it | Slightly familiar | Not familiar at all | |
| 1. **Impact of blood eosinophil count on glucocorticoid treatment in COPD patients.** | Very familiar | Fairly familiar | Heard of it | Slightly familiar | Not familiar at all | |
| 1. **Correct use and precautions for inhaled corticosteroids (ICS).** | Very familiar | Fairly familiar | Heard of it | Slightly familiar | Not familiar at all | |
| 1. **Strategies for tapering and discontinuation of inhaled corticosteroids (ICS).** | Very familiar | Fairly familiar | Heard of it | Slightly familiar | Not familiar at all | |
| 1. **Strategies for tapering and discontinuation of systemic corticosteroids (SCS).** | Very familiar | Fairly familiar | Heard of it | Slightly familiar | Not familiar at all | |

| **Part 3 Attitudes Toward the Use of Glucocorticoids in COPD** | | | | | |
| --- | --- | --- | --- | --- | --- |
| 1. **I believe inhaled corticosteroids (ICS) are necessary in the treatment of stable COPD.** | a. strongly agree | b. agree | c. neutral | d. disagree | e. strongly disagree |
| 1. **I believe systemic corticosteroids (SCS) are necessary in the treatment of acute exacerbations of COPD.** | a. strongly agree | b. agree | c. neutral | d. disagree | e. strongly disagree |
| 1. **I believe the use of glucocorticoids should strictly follow guidelines and expert consensus.** | a. strongly agree | b. agree | c. neutral | d. disagree | e. strongly disagree |
| 1. **I believe it is necessary to regularly evaluate the treatment regimen of inhaled corticosteroids (ICS) in COPD patients.** | a. strongly agree | b. agree | c. neutral | d. disagree | e. strongly disagree |
| 1. **I support adjusting the use of inhaled corticosteroids (ICS) based on blood eosinophil counts.** | a. strongly agree | b. agree | c. neutral | d. disagree | e. strongly disagree |
| 1. **I believe ICS use should be gradually reduced or discontinued in patients without clear indications.** | a. strongly agree | b. agree | c. neutral | d. disagree | e. strongly disagree |
| 1. **I believe the risks of long-term glucocorticoid use in COPD patients outweigh the benefits.** | a. strongly agree | b. agree | c. neutral | d. disagree | e. strongly disagree |
| 1. **I believe individualized treatment for COPD patients is very important.** | a. strongly agree | b. agree | c. neutral | d. disagree | e. strongly disagree |
| 1. **I believe patients often have misconceptions about glucocorticoids, particularly regarding their short-term and long-term adverse effects.** | a. strongly agree | b. agree | c. neutral | d. disagree | e. strongly disagree |
| 1. **I believe patient education is an important component of both inhaled and systemic glucocorticoid use.** | a. strongly agree | b. agree | c. neutral | d. disagree | e. strongly disagree |
| 1. **I believe collaboration within the medical team is very important for glucocorticoid use in COPD patients.** | a. strongly agree | b. agree | c. neutral | d. disagree | e. strongly disagree |
| 1. **I believe hospitals or departments should organize more educational sessions on the use of glucocorticoids in COPD.** | a. strongly agree | b. agree | c. neutral | d. disagree | e. strongly disagree |

| **Part 4 Practices Regarding the Use of Glucocorticoids in COPD**  Always: almost 100%; Often: ≥70%; Sometimes: ~40–70%; Rarely: ~10–40%; Never: <10% or never at all | | | | | |
| --- | --- | --- | --- | --- | --- |
| 1. **I regularly re-evaluate glucocorticoid treatment regimens for COPD patients.** | a. always | b. often | c. sometimes | d. rarely | e. never |
| 1. **I provide individualized glucocorticoid treatment for COPD patients based on the latest guidelines and expert consensus.** | a. always | b. often | c. sometimes | d. rarely | e. never |
| 1. **I adjust the use of inhaled corticosteroids (ICS) based on patients’ blood eosinophil counts.** | a. always | b. often | c. sometimes | d. rarely | e. never |
| 1. **I routinely use systemic corticosteroids (SCS) during acute exacerbations of COPD.** | a. always | b. often | c. sometimes | d. rarely | e. never |
| 1. **I explain in detail to COPD patients the correct use and precautions of inhaled corticosteroids (ICS).** | a. always | b. often | c. sometimes | d. rarely | e. never |
| 1. **I proactively educate patients and their families about the potential risks of glucocorticoid use during COPD treatment.** | a. always | b. often | c. sometimes | d. rarely | e. never |
| 1. **I closely monitor any abnormalities or adverse reactions during patients’ medication use.** | a. always | b. often | c. sometimes | d. rarely | e. never |
| 1. **I regularly study the latest guidelines and expert consensus.** | a. always | b. often | c. sometimes | d. rarely | e. never |
